# A simple and efficient technique for suturing and knotting during endoscopic dacryocystorhinostomy

**DOI:** 10.1007/s10792-022-02388-1

**Published:** 2022-07-15

**Authors:** Rongxin Chen, Shu Liu, Aixin Jiang, Aizezi Wumaier, Yuanxia Yang, Xinyue Yu, Ziwei Meng, Yuxiang Mao, Xuanwei Liang

**Affiliations:** 1 State Key Laboratory of Ophthalmology, Zhongshan Ophthalmic Center, Sun Yat-sen University, Guangdong Provincial Key Laboratory of Ophthalmology and Visual Science, Guangdong Provincial Clinical Research Center for Ocular Diseases, Sun Yat-Sen University, Guangzhou, 510060 China; 2 State Key Laboratory of Oncology in South China, Collaborative Innovation Center for Cancer Medicine, Sun Yat-Sen University Cancer Center, Guangzhou, 510060 China; 3 Ophthalmologic Center, First People’s Hospital of Kashi Prefecture, Affiliated Kashi Hospital of Sun Yat-Sen University, Kashi, 844000 China

**Keywords:** Endoscopic, Dacryocystorhinostomy, Nasolacrimal duct obstruction, Outcomes, Suturing and knotting

## Abstract

**Purpose:**

This study evaluated the long-term outcomes of endoscopic suturing and knotting-dacryocystorhinostomy (eSK-DCR) without the use of a stent or mitomycin C.

**Methods:**

A prospective interventional case series was performed on patients with nasolacrimal duct obstruction (NLDO) who underwent eSK-DCR at Zhongshan Ophthalmic Center from October 2019 to December 2019. The surgeon sutured the lacrimal sac mucosa with the nasal mucosa by tying knots under endoscopic DCR. Subject demographics, preoperative data and postoperative data were collected, including clinical presentation, Munk score for epiphora, surgical indications, operation time, duration of knotting, number of knots, endoscopic ostium size, complications, and anatomical and functional success. Anatomic success was defined as patent ostium on lacrimal irrigation, and functional success was defined as subjective improvement in symptoms. Statistical analysis was performed by IBM SPSS software (Version 20.0; SPSS Inc., Chicago, IL, USA).

**Results:**

A total of 60 patients (71 eyes) underwent pure eSK-DCR. Of these, 95.0% (57/60) were females. The mean age of the patients was 54.7 years. The mean surgical time was 37.60 min, and the average time for each knotting was 2.86 min. Endoscopic evidence found that all patients showed patent ostium and normal healing of the flaps after 4 weeks. The Munk scores dropped significantly at 6 months postoperatively compared to preoperative scores (*P* < 0.0001). Although 4 patients (7 eyes) were lost to follow-up at the end of the 2-year period, the anatomical and functional success remained stable during the 2-year follow-up period (anatomical, 100%; functional, 87.5%). No serious complications were detected during the follow-up period.

**Conclusion:**

Pure eSK-DCR is a simple and reliable therapeutic method for the management of NLDO. The surgical outcomes were good and remained stable beyond 6 months postoperatively.

**Supplementary Information:**

The online version contains supplementary material available at 10.1007/s10792-022-02388-1.

## Introduction

Since the end of the twentieth century, there has been a shift toward accepting endoscopic dacryocystorhinostomy (DCR) as being as safe and effective as external DCR for the treatment of patients with nasolacrimal duct obstruction (NLDO). Endoscopic DCR has very high success rates [1]; however, there are instances in which the procedure fails, mainly due to anatomic obstruction by granulation tissue, synechia between the lateral wall and the middle turbinate, and membrane closure of the neo-ostium during healing, regardless of the surgical approach [2–4]. Different intraoperative adjunctive procedures have been used to increase the success rate of endoscopic DCR, including topical mitomycin C (MMC) [5, 6], stent intubation [7], and postoperative packing with or without medication, e.g., topical steroids [8].

The principles of enhanced endoscopic DCR techniques mimic those of external DCR, mainly by providing adequate bone ostium, mucosal flaps, and mucosal flap apposition, and thereby aiming for primary intention healing and minimizing soft tissue scarring. Moreover, it is important to avoid abnormal ostial healing induced by false localization of the lacrimal sac and nasal mucosal flaps [2]. To predict the pattern of anastomosis [9], the suturing technique with mucosal flaps in endoscopic DCR was first described by Kirtane et al. [10] and Tachino et al. [11]. However, their technique was not widely accepted because they also used MMC or tube intubation and a vascular clip was also needed during their endoscopic DCR technique. MMC use was reported to have no effects on the long-term results of patients undergoing endoscopic DCR [12]. Furthermore, granulation tissue formation around the stent tubes is still a problem that is encountered during endoscopic procedure [13]. Routine intubation in endoscopic DCR is not necessary for patients with NLDO because no significant difference was found whether intubation was used [14, 15]. Therefore, the appropriate surgery technique is vital for successful results, especially for mucosal edge-to-edge apposition.

For the above reasons, we introduced a pure suturing and knotting technique, which we named endoscopic suturing and knotting-dacryocystorhinostomy (eSK-DCR), for intranasal suturing of mucosal flaps under tension. This technique allows the ophthalmic surgeon to perform the knot without a special instrument; therefore, it is a convenient and cost-effective approach. It is easy to learn this technique, which ensures secure and tensional knots for mucosal flaps. The main objective of this study is to investigate the efficiency of the pure suturing and knotting technique for endoscopic DCR and to describe its clinical practice.

## Methods

Patients with NLDO, who visited the clinic of an oculoplastic surgeon (X.L.) and required DCR were invited to participate in this prospective, nonrandomized noncomparative interventional study from October 2019 to December 2019. The exclusion criteria included: patients under 18 years of age, previous history of DCR, coexisting lacrimal disorders, such as punctual and canalicular stenosis, mucoceles, and coexisting nasal disorders such as deviated septum, concha bullosa, ectropion, and facial palsy. Patients who received topical antiglaucoma medications, underwent laser lacryocystoplasty, had orbital trauma and received radiotherapy were also excluded. The parameters that were studied included patient demographics, clinical presentation, significant past histories, minimum length of dacryocystography, duration of surgery, duration of knotting, number of knots, pre- and postoperative Munk scale scores [16], endoscopic ostium size, anatomical and functional success, and complications.

This study was approved by the Ethics Committee of Zhongshan Ophthalmic Center, Sun Yat-sen University (No. 2021KYPJ100). Informed consent was obtained from each patient in accordance with the Declaration of Helsinki.

### Surgical procedures

**Fig. 1:**
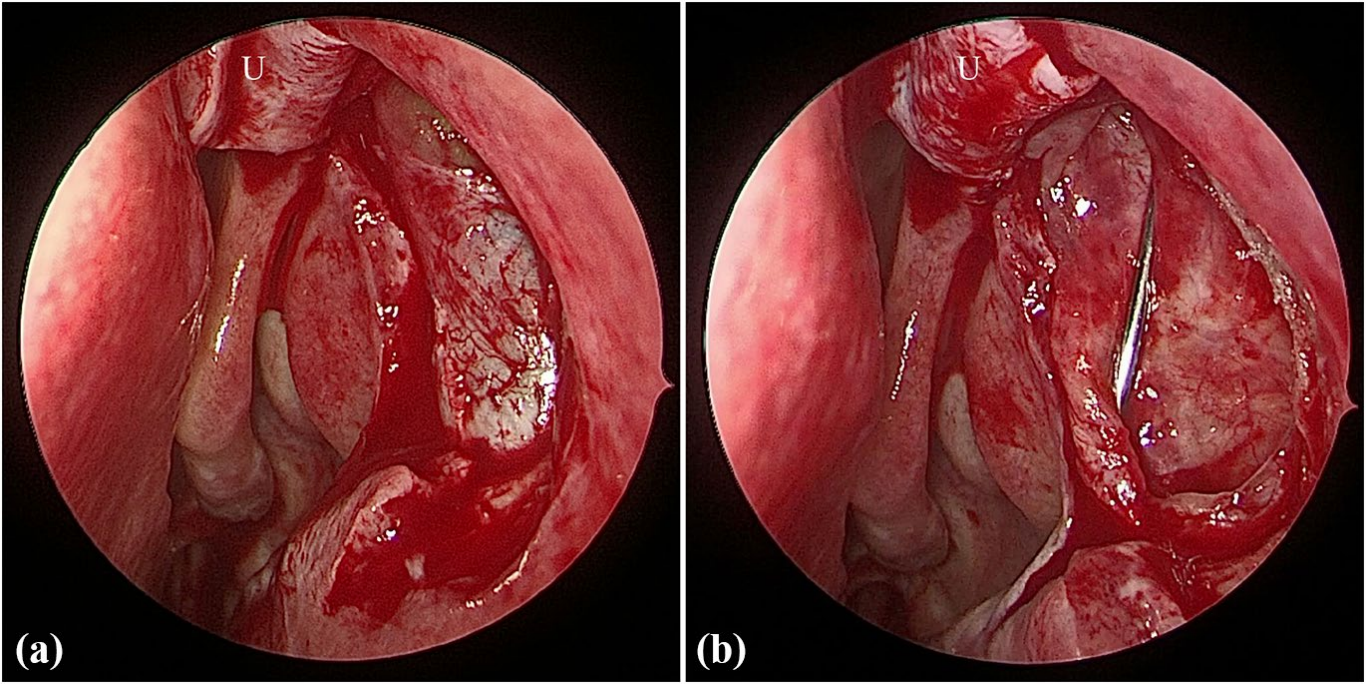
Typical clinical photograph of the endoscopic dacryocystorhinostomy procedure. **a** After opening the bony ostium, the lacrimal sac was exposed. **b** The lacrimal sac flap was lifted and then reflected onto the residual nasal mucosa. U = upper U-shape flap

**Fig. 2:**
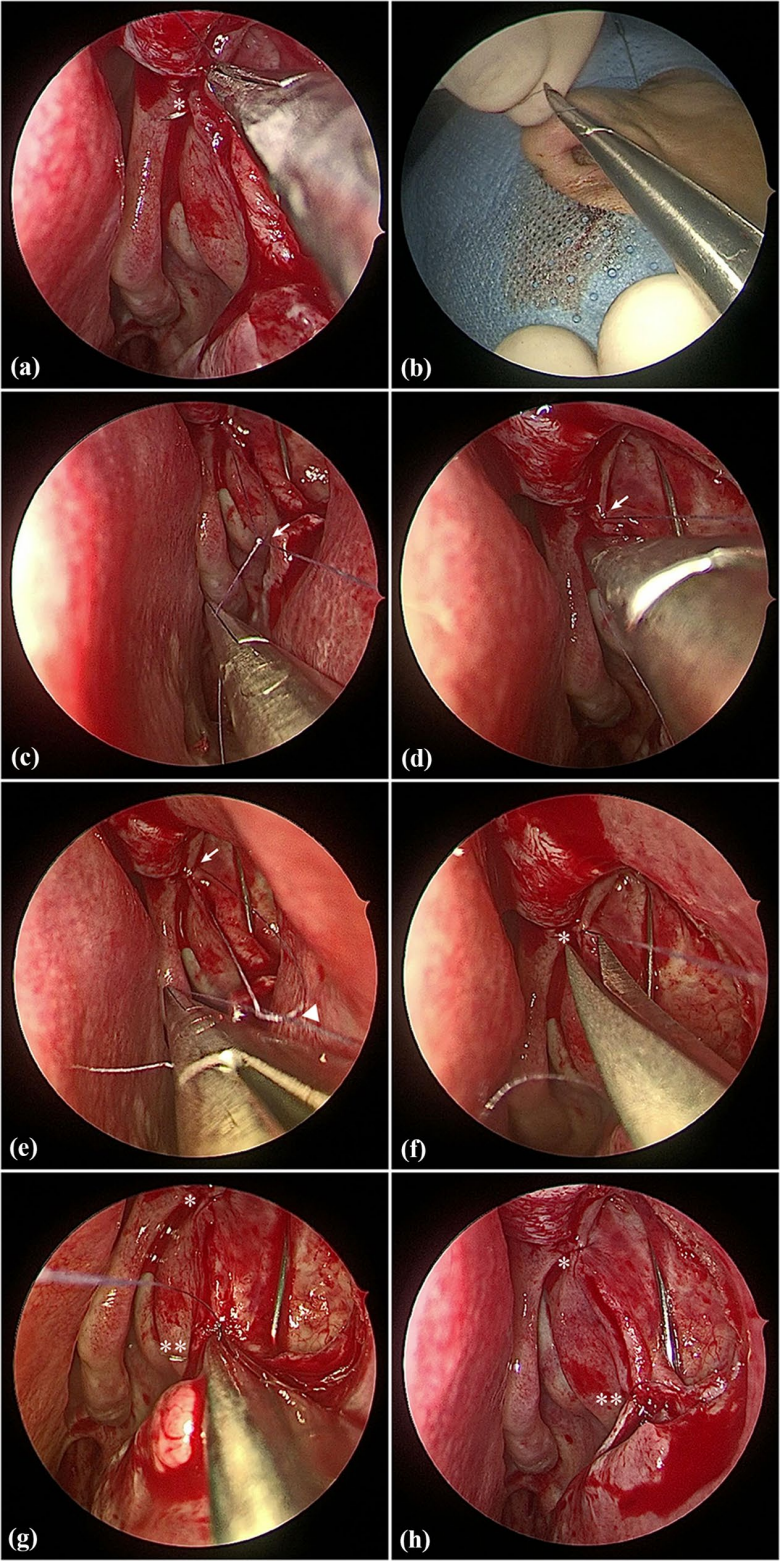
Suturing and knotting technique for endoscopic dacryocystorhinostomy. **a** Microneedle insertion through the lacrimal and nasal mucosa flaps under endoscopy. **b** The first knot was made outside of the nostril. **c** One end of the thread was grasped using the ophthalmic needle holder, and the first knot was slide through the anterior naris. **d** The first knot was placed close to the nasal mucosa, while tension was maintained on both threads. **e** After making a second knot outside the nostril, a second knot was made and placed to tighten the first knot using the ophthalmic needle holder while tension was maintained on both threads. **f** The excess suture filaments of the first suture were cut off. **g** A second suture was performed using the same technique as (**a**-**f**). **h** After suturing and knotting, the lacrimal flap was accurately anastomosed to the nasal mucosa flap. Triangle = first knot on the mucosa; arrow = second knot; asterisk = first suture; double asterisk = second suture

All surgeries were performed under general anesthesia by the same experienced surgeon (X. L.). The surgical procedures of nasal mucosa and bone ostium were then performed as described in our previous report [17]. After the lacrimal sac was fully exposed (Fig. 1a), the upper or lower canaliculus was probed to tent the medial wall of the lacrimal sac. Ultimately, the medial lacrimal sac mucosa was incised to form a door “]”-shaped flap, and this posteriorly based flap was then reflected onto the residual nasal mucosa (Fig. 1b). Figure 2 demonstrates how to place the sutures and knots after creating the lacrimal flaps. A microneedle with 7–0 Vicryl sutures (Ethicon, USA) was inserted through the lacrimal sac and nasal mucosal edges under an endoscopic intranasal approach (Fig. 2a), and the first knot was made manually outside the nostril (Fig. 2b). Next, one end of the suture filament was held by an assistant, and the other end was held by an ophthalmic needle holder, which stayed in the surgeon’s dominant hand, and a knot was then slid into the nasal cavity (Fig. 2c). While keeping tension on both strings, the first knot, located close to the mucosa, was grasped by the ophthalmic needle holder and tightened to the mucosa (Fig. 2d). Additionally, a second knot was made outside of the nostril, slid onto the mucosal surface (Fig. 2e), and firmly tightened. Finally, the excess suture filaments were cut off at the end (Fig. 2f). The same procedure (Fig. 2g) of suturing and knot tying was performed for the accurate apposition of the mucosal flaps (Fig. 2h) as needed. After tying the knot, the redundancy of the U-shaped flap was trimmed to cover the upper bony ostium, and nasal tamponade was placed at the osteotomy site. Further details of the suturing and knotting technique are demonstrated in Video S1.

Postoperatively, all patients were prescribed a nasal steroid spray and antibiotic eye drops that were applied at home, followed by intranasal irrigations and saline spray applications that were performed to prevent crust formation. Beyond 4 weeks after surgery, the nasal tamponade and crusts were removed, and the size and patency of the newly created ostium were checked using endoscopic evaluation. We also divided the ostium size (OS) into four groups: large (> 4 mm), medium (2–4 mm), small (< 2 mm) of minimal diameter, and obstruction (0 mm). The OS measurement was performed using a 2 mm olive tip suction device.

Postoperative follow-up visits were scheduled for 6 months and 2 years. Anatomical success was defined as patent ostium on lacrimal irrigation. All the cases were asked to compare their pre- and postoperative complaints according to a 5-point Likert scale: no complaints of epiphora or cure (4-score), significant improvement (symptoms are at least 80% better when compared with preoperative symptoms) (3-score), moderate/slight improvement (2-score), unchanged (1-score), and worsening (0-score) in the symptoms. Functional success was defined as subjective improvement in symptoms with a 4-score or 3-score. The secondary outcome was the presence of complications, including orbital fat prolapse, postoperative bleeding, granulomas threatening the ostium, nasal synechiae, and false localization of the mucosal flaps.

### Statistical analysis

The statistical analysis was performed using IBM SPSS software (Version 20.0; SPSS Inc., Chicago, IL, USA). The compliance of distributions with the normal distribution was tested using the Shapiro–Wilk test. Given that the assumptions concerning the use of parametric methods were not met, the statistical hypotheses were verified with nonparametric methods. The methods used were the chi-square test, Kruskal–Wallis test, or one-way ANOVA on ranks. Bonferroni correction was applied for multiple comparisons. A P-value of < 0.05 was considered statistically significant.

## Results

A total of 63 eyes of 53 patients with NLDO who underwent eSK-DCR were studied. Of these, 18.9% (10/53) underwent bilateral surgery simultaneously. The male-to-female ratio was 2:51. The age of the patients at the time of surgery varied from 26.0 to 82.0 years, with a mean age (± SD) of 55.3 ± 12.2 years. The indications for surgery and significant past histories are summarized in Table 1. Of these patients, epiphora with discharge was a universal presentation, with a ratio of 73.6% (47 eyes of 39 patients). The mean operating time was 36.7 ± 14.48 min. The mean number of sutures and knots was 1.32 ± 0.50 per eye, and the average time per knot was 2.80 ± 0.89 min.

**Table 1:** Indications for surgery and significant past histories

	No. of eyes (%)
Indications for surgery	
Chronic dacryocystitis	67 (94.4)
Acute dacryocystitis	4 (5.6)
Significant past histories	
Nasolacrimal probing	22 (34.9)
Nasolacrimal intubation	9 (14.3)
Orbital surgery	1 (1.6)
Haemophagocytic syndrome	1 (1.6)

After a mean period of 46.2 ± 12.8 days (range, 28 to 70 days), all patients had endoscopic evidence of a patent ostium with common canalicular openings assessed by lacrimal syringing. Overall, 11.1%, 63.5% and 25.4% of the eyes had large, medium and small OS, respectively.

**Fig. 3:**
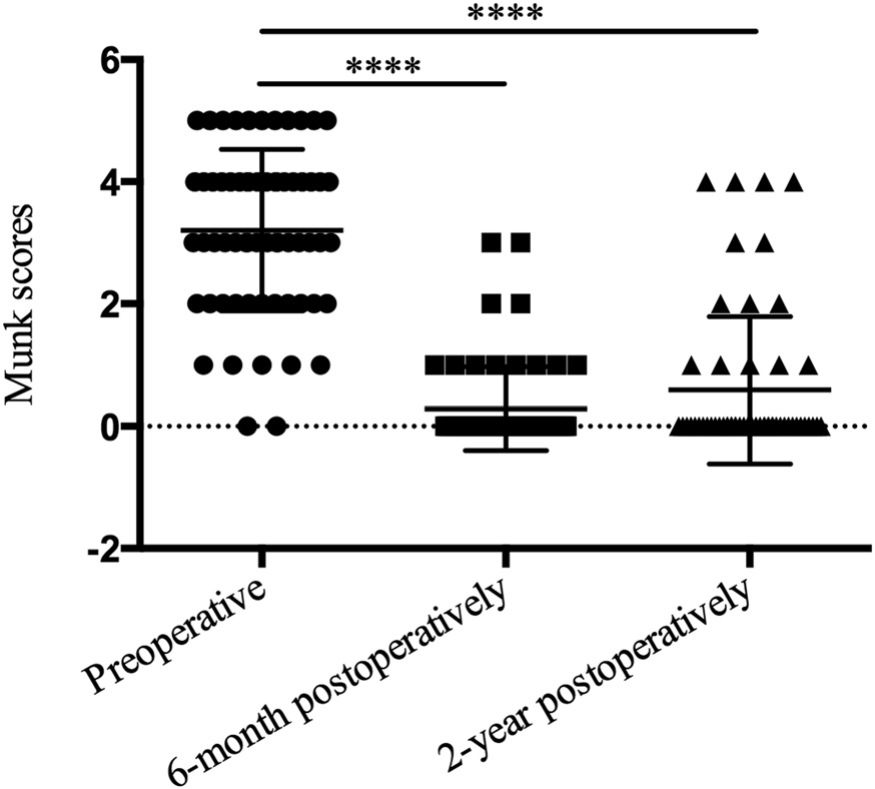
Munk scores of epiphora. Subjects were able to achieve a reduction in eSK-DCR performed. Subjects averaged 3.21 scores (± 1.32 standard deviation) preoperatively. They averaged 0.29 scores (± 0.68 standard deviation) at postoperative month 6 and 0.59 scores (± 1.20 standard deviation) at postoperative year 2. The Kruskal–Wallis test comparing preoperative and postoperative month 6 or postoperative year 2 scores showed all *P* < 0.0001

The Munk scores were decreased significantly at 6 months and 2 years postoperatively compared with preoperative scores, and with mean scores of 0.29 ± 0.68, 0.59 ± 1.20, and 3.21 ± 1.32, respectively (Fig. 3). As shown in Table 2, eSK-DCR produced good anatomical and functional success rates at 6 months postoperatively, and the rates were maintained until the 2-year follow-up (anatomical, 100%; functional, 87.5%; all *P* > 0.05), although 4 patients (7 eyes) were lost to follow-up at the end of 2 years.

**Table 2:** Postoperative anatomical and functional success of eSK-DCR

Total number of eyes	Post-op	Anatomical success		Functional success	
		n (%)	*P* value	n (%)	*P* value
63	6 months	63 (100)	1.00	59 (93.7)	0.248
56	2 years	56 (100)		49 (87.5)	

There were 3 patients with periosteal granulomas, which did not threaten the ostium. No other complications arising from the use of eSK-DCR were observed in the present cases, thereby, confirming the safety of this technique.

## Discussion

Endoscopic DCR is now suggested as a well-established alternative to external DCR [18]; however, there is difficulty in maintaining mucosal anastomosis under an endoscopic approach. False approximation of the mucosa is one of the reasons for the development of rhinostomy closure after endoscopic DCR. The current study describes the long-term outcomes and complications of our described endoscopic DCR technique. Highly successful outcomes were observed after following the patients beyond 6 months, and the outcomes were maintained during the 2-year follow-up period of our study on the simple knotting technique, named eSK-DCR, which describes suturing for mucosal apposition under tension during an endoscopic intranasal approach.

According to previous reports [19–21], the majority of ostium shrinkage occurs in the first 4 weeks after surgery; thereafter, the OS appears to be stable with a lesser degree of shrinkage between 1 and 12 months postoperatively. The knotting technique that was applied during endoscopic DCR achieved good outcomes compared with external DCR, and the approximation of mucosal edge-edge results in primary intention healing with less bone exposure, minimal granulation tissue and closure of the postoperative ostium. Moreover, ostial closure and false localization of the mucosa did not occur in any eye that was included in the current study beyond 4 weeks postoperatively. The previous study reported that patients who underwent external DCR had a mean ostium width of 3.60 ± 2.24 mm [22], and more than half of patients obtained an ostium with diameter of 2 to 4 mm in the current study. An anatomically patent ostium is definitely required during endoscopic DCR, however, it is believed that ostium size does not affect functional outcomes [22, 23]. As reported in our study, the anatomical success rate was higher than the functional success rate. Failure seems to be multifactorial and should be considered in older, smoking, male patients and those with multipathological diagnoses, with a long duration of the condition, even with good anatomical results [24, 25].

Another important aspect of endoscopic DCR is how we measure surgical outcomes and means to compare the successes of different techniques. The Royal College of Ophthalmologists (1999) published guidelines for clinical governance suggesting that freedom from epiphora 3 months after surgery is a marker for a satisfactory procedure [26]. In our study, the significant decrease in postoperative Munk scores of epiphora suggested that results were stable at the 6-month follow-up, and no further significant changes in the outcome were observed beyond 2 years. Schlachter et al. [27] reported that subjective complaints of epiphora correlated with patency of the nasolacrimal system 3 months postoperatively. We therefore used significant improvement or free symptoms postoperatively as the measure of functional success. The eSK-DCR had a functional success rate of 87.5%, which was consistent with previous reports of endoscopic DCR [89.5% (87.2–91.5%)] [18]. Based on the above evidence, we deduced that eSK-DCR is an efficient technique for patients with NLDO.

For patients with no canalicular disease, there is no evidence in the existing reports showing that stent intubation during endoscopic DCR is superior to nonintubation [14, 28]. Other studies [29–32] have reported that intubation itself may cause granulation formation, predisposing infection, punctal lacerations, patient discomfort, and increased costs related to intubation and even lead to surgical failure in the long run. Silicone stents can be inserted during surgery unless there is canalicular scarring, a large valve of Rosenmuller occluding the common canaliculus, or a tight common canaliculus opening. The surgical success rates were similar in the condition of endoscopic DCR with or without tubing [31]. Likewise, for the current eSK-DCR series with no stent use, there were no cases of canalicular closure or stenosis for NLDO postoperatively. If the mucosal flaps created intraoperatively can be well apposed for mucosal anastomosis under eSK-DCR, we recommend that routine intubation to maintain the ostial opening is not necessary.

MMC is an antiproliferative agent and prevents postoperative adhesions and scarring around the ostium. The role of MMC has been reported in several studies. Kirtane et al. [10] showed an endoscopic DCR technique with a primary success rate of 95% for NLDO. Apart from using MMC, they emphasized suturing or vascular clips to join the lacrimal sac wall and mucosal flap together. However, the knotting and suturing technique created a mucosalized surface that potentially prevented the formation of synechia and granulation tissue around the tube and contraction of the opening fistula. We therefore tended to not use MMC in all our cases.

Consumable costs are an important factor in health insurance. The eSK-DCR technique avoided the extra medical costs related to lacrimal intubation or MMC, and ophthalmic conventional instruments were used to perform endoscopic knot tying rather than costly technique-specific instruments. Moreover, operative time is an important factor in the intraoperative safety of patients, and a prolonged operative time can increase the risk of surgery and complications. Based on 2.86 ± 0.89 min per knotting of the current study, this time that has been used can potentially be performed by surgeons, and it would increase the popularity of eSK-DCR with a simple technique and low cost.

The benefit of eSK-DCR is the accurate apposition of the lacrimal flaps and nasal mucosa during endoscopic DCR. However, the disadvantage of eSK-DCR is that extra time must be allowed for knotting and suturing. This extra time will be reduced as surgeons gain experience in the suturing and knotting technique. Moreover, conventional endoscopic DCR is widely practised, and eSK-DCR can be easily adopted by those with experience in conventional endoscopic DCR. This suturing and knotting technique based on surgical principles is similar to that of an external DCR. Therefore, the success rate of eSK-DCR is comparable to that of external DCR. The knotting and suturing procedure can be performed using existing ophthalmic instruments with no need for new specialized tools; therefore, eSK-DCR has potential value to be popularized throughout the world and would be advantageous to ophthalmologists who perform endoscopic DCR.

Our study has limitations, such as the lack of a direct comparison between eSK-DCR and conventional endoscopic DCR. Further prospective comparative trials are anticipated. We used lacrimal irrigations and subjective evaluation to assess surgical success, which closely mirrors endoscopic evidence for ostium patency [27]. However, it is possible that the failure rate was underestimated by the lack of long-term postoperative endoscopic follow-up.

In conclusion, the current study has demonstrated that pure eSK-DCR for patients with NLDO is a simple, effective and safe technique, based on stable anatomical apposition.

## Supplementary Information

Below is the link to the electronic supplementary material.

Supplementary file1 (DOCX 28 KB)

## Declarations

### Availability of Data and Material

The data used for the analysis are available from the corresponding author upon reasonable request.

## References

[1] Marcet MM, Kuk AK, Phelps PO (2014). Evidence-based review of surgical practices in endoscopic endonasal dacryocystorhinostomy for primary acquired nasolacrimal duct obstruction and other new indications. Curr Opin Ophthalmol.

[2] Onerci M, Orhan M, Ogretmenoğlu O, Irkeç M (2000). Long-term results and reasons for failure of intranasal endoscopic dacryocystorhinostomy. Acta Oto-Laryngol.

[3] Baek JS, Jeong SH, Lee JH, Choi HS, Kim SJ, Jang JW (2017). Cause and management of patients with failed endonasal dacryocystorhinostomy. Clin Exp Otorhinolaryngol.

[4] Lin GC, Brook CD, Hatton MP, Metson R (2017). Causes of dacryocystorhinostomy failure: external versus endoscopic approach. Am J Rhinol Allergy.

[5] Selig YK, Biesman BS, Rebeiz EE (2000). Topical application of mitomycin-C in endoscopic dacryocystorhinostomy. Am J Rhinol.

[6] Do JR, Lee H, Baek S, Lee TS, Chang M (2016). Efficacy of postoperative mitomycin-C eye drops on the clinical outcome in endoscopic dacryocystorhinostomy. Graefe's Arch Clin Exp Ophthalmol.

[7] Wulfman DR, Harrison AR, Hultman D (2005). Novel stent for dacryocystorhinostomy (DCR) and other surgical applications. J Biomech Eng.

[8] Chong KK, Lai FH, Ho M, Luk A, Wong BW, Young A (2013). Randomized trial on silicone intubation in endoscopic mechanical dacryocystorhinostomy (SEND) for primary nasolacrimal duct obstruction. Ophthalmology.

[9] Yuen KS, Lam LY, Tse MW, Chan DD, Wong BW, Chan WM (2004). Modified endoscopic dacryocystorhinostomy with posterior lacrimal sac flap for nasolacrimal duct obstruction. Hong Kong Med J.

[10] Kirtane MV, Lall A, Chavan K, Satwalekar D (2013). Endoscopic dacryocystorhinostomy with flap suturing. Indian J Otolaryngol Head Neck Surg.

[11] Tachino H, Fujisaka M, Fuchizawa C, Tsubota M, Takakura H, Ishida M, Hayashi A, Shojaku H (2015). Endonasal flap suture-dacryocystorhinostomy (eFS-DCR): a new surgical technique for nasolacrimal duct obstruction (NLDO). Acta Oto-Laryngol.

[12] Prasannaraj T, Kumar BYP, Narasimhan I, Shivaprakash KV (2012). Significance of adjunctive mitomycin C in endoscopic dacryocystorhinostomy. Am J Otolaryngol.

[13] Borlingegowda V, Vijayashree MS (2015). Silicone stenting and polypropylene stenting in endoscopic dacryocystorhinostomy: a prospective comparative study. Res Otolaryngol.

[14] Cannon PS, Chan W, Selva D (2013). Incidence of canalicular closure with endonasal dacryocystorhinostomy without intubation in primary nasolacrimal duct obstruction. Ophthalmology.

[15] Chin J, Lam V, Chan R, Li CL, Yeung L, Law A, Young A, Yuen H, Ali MJ, Chong KKL (2020). Comparative study of stenting and ostium packing in endoscopic dacryocystorhinostomy for primary acquired nasolacrimal duct obstruction. Sci Rep.

[16] Munk PL, Lin DT, Morris DC (1990). Epiphora: treatment by means of dacryocystoplasty with balloon dilation of the nasolacrimal drainage apparatus. Radiology.

[17] Peng W, Tan B, Wang Y, Wang H, Wang Z, Liang X (2017). A modified preserved nasal and lacrimal flap technique in endoscopic dacryocystorhinostomy. Sci Rep.

[18] Vinciguerra A, Nonis A, Giordano Resti A, Bussi M, Trimarchi M (2020). Best treatments available for distal acquired lacrimal obstruction: a systematic review and meta**-**analysis. Clin Otolaryngol.

[19] Chan W, Selva D (2013). Ostium shrinkage after endoscopic dacryocystorhinostomy. Ophthalmology.

[20] Ali MJ, Psaltis AJ, Ali MH, Wormald PJ (2015). Endoscopic assessment of the dacryocystorhinostomy ostium after powered endoscopic surgery: behaviour beyond 4 weeks. Clin Exp Ophthalmol.

[21] Mann BS, Wormald PJ (2006). Endoscopic assessment of the dacryocystorhinostomy ostium after endoscopic surgery. Laryngoscope.

[22] Torun MT, Yılmaz E (2021). The role of the rhinostomy ostium size on functional success in dacryocystorhinostomy. Braz J Otorhinolaryngol.

[23] Al Huthail RR, Al-Faky YH (2021). Late endoscopic evaluation of the ostium size after external dacryocystorhinostomy. Eur J Ophthalmol.

[24] García Callejo FJ, Juantegui Azpilicueta M, Balaguer García R (2022). Factors involved in the success and failure of endoscopic dacryocystorhinostomy from our experience. Acta Otorrinolaringol Esp (Engl Ed).

[25] Cohen O, Amos I, Halperin D, Bavnik Y, Milstein A, Shoshani Y, Leiba H, Warman M (2021). Five- and 10-year outcomes for primary endoscopic dacryocystorhinostomy: failure rate and risk factors. Laryngoscope.

[26] Yung MW, Hardman-Lea S (2002). Analysis of the results of surgical endoscopic dacryocystorhinostomy: effect of the level of obstruction. Br J Ophthalmol.

[27] Schlachter DM, Richani K, Black EH (2016). Diode laser-assisted endocanalicular dacryocystorhinostomy: a prospective study. Ophthalmic Plast Reconstr Surg.

[28] Kim DH, Kim SI, Jin HJ, Kim S, Hwang SH (2018). The clinical efficacy of silicone stents for endoscopic dacryocystorhinostomy: a meta-analysis. Clin Exp Otorhinolaryngol.

[29] Saeed BMN (2012). Endoscopic DCR without stents: clinical guidelines and procedure. Eur Arch Oto-Rhino-Laryngol.

[30] Unlu HH, Gunhan K, Baser EF, Songu M (2009). Long-term results in endoscopic dacryocystorhinostomy: is intubation really required?. Otolaryngol Head Neck Surg.

[31] Al-Qahtani AS (2012). Primary endoscopic dacryocystorhinostomy with or without silicone tubing: a prospective randomized study. Am J Rhinol Allergy.

[32] Allen K, Berlin AJ (1989). Dacryocystorhinostomy failure: association with nasolacrimal silicone intubation. Ophthalmic Surg.

