# Supplementary material for: A simple and efficient technique for suturing and knotting during endoscopic dacryocystorhinostomy
Source: Int Ophthalmol. 2022 Jul 15;43(1):63–71. doi: 10.1007/s10792-022-02388-1 (PMC9902408; doi:10.1007/s10792-022-02388-1)
Supplement: Supplementary file 1 — Supplementary file1 (DOCX 28 KB) [file 10792_2022_2388_MOESM1_ESM.docx]

**Supplementary Materials**

Video S1. Further details of the suturing and knotting technique during endoscopic DCR.
